# Role of Cystic Fibrosis Bronchial Epithelium in Neutrophil Chemotaxis

**DOI:** 10.3389/fimmu.2020.01438

**Published:** 2020-08-04

**Authors:** Giulio Cabrini, Alessandro Rimessi, Monica Borgatti, Ilaria Lampronti, Alessia Finotti, Paolo Pinton, Roberto Gambari

**Affiliations:** ^1^Center for Innovative Therapies in Cystic Fibrosis, University of Ferrara, Ferrara, Italy; ^2^Department of Life Sciences and Biotechnology, University of Ferrara, Ferrara, Italy; ^3^Department of Neurosciences, Biomedicine and Movement, University of Verona, Verona, Italy; ^4^Department of Medical Sciences, University of Ferrara, Ferrara, Italy

**Keywords:** cystic fibrosis, epithelium, lung, chemotaxis, neutrophil, inflammation

## Abstract

A hallmark of cystic fibrosis (CF) chronic respiratory disease is an extensive neutrophil infiltrate in the mucosa filling the bronchial lumen, starting early in life for CF infants. The genetic defect of the CF Transmembrane conductance Regulator (CFTR) ion channel promotes dehydration of the airway surface liquid, alters mucus properties, and decreases mucociliary clearance, favoring the onset of recurrent and, ultimately, chronic bacterial infection. Neutrophil infiltrates are unable to clear bacterial infection and, as an adverse effect, contribute to mucosal tissue damage by releasing proteases and reactive oxygen species. Moreover, the rapid cellular turnover of lumenal neutrophils releases nucleic acids that further alter the mucus viscosity. A prominent role in the recruitment of neutrophil in bronchial mucosa is played by CF bronchial epithelial cells carrying the defective CFTR protein and are exposed to whole bacteria and bacterial products, making pharmacological approaches to regulate the exaggerated neutrophil chemotaxis in CF a relevant therapeutic target. Here we revise: (a) the major receptors, kinases, and transcription factors leading to the expression, and release of neutrophil chemokines in bronchial epithelial cells; (b) the role of intracellular calcium homeostasis and, in particular, the calcium crosstalk between endoplasmic reticulum and mitochondria; (c) the epigenetic regulation of the key chemokines; (d) the role of mutant CFTR protein as a co-regulator of chemokines together with the host-pathogen interactions; and (e) different pharmacological strategies to regulate the expression of chemokines in CF bronchial epithelial cells through novel drug discovery and drug repurposing.

## Lung Pathology in Cystic Fibrosis Patients: an Early Event Accompanying Whole Life

Autosomal recessive inheritance of mutations of the Cystic Fibrosis Transmembrane Conductance Regulator (*CFTR*) gene, encoding a chloride and bicarbonate transporting protein, is at the basis of the multiorgan Cystic Fibrosis (CF) disease (1–3). CF lung disease, characterized by chronic bacterial airway infection, neutrophilic inflammation, and dilation of bronchioles obstructed by mucus plugs, is presently the main limitation to the quality and expectancy of the life of CF patients. Although lung pathology and the mechanisms of the disease were prioritized for decades in CF research, what is between the *CFTR* gene defects and the overt clinical symptoms of the CF patients has still not been completely defined. Consensus has been reached that lung pathology begins in the early months of life for the majority of CF infants, often before the onset of clinical symptoms, as demonstrated by the presence of inflammatory cytokines in the bronchoalveolar lavage fluid of CF infants (4–6) and by the lung histopathology of CF infants who die within weeks or months after birth, showing bronchial lumena filled and plugged by neutrophils (7).

Different hypotheses have been proposed to link the chloride and bicarbonate transport defects of mutant CFTR protein and the onset of airway disease. Consensus on the mechanism can be summarized in that altered CFTR protein reduces the hydration, and possibly the pH, of the airway surface liquid (ASL), thus affecting the rate of the mucociliary clearance, the principal innate mechanism involved in the defense against microbial infection (8). ASL dehydration worsens the mucociliary clearance by reducing mucus fluidity in both ASL and in the submucosal glands of the airway mucosa. The precise mechanism(s) favoring the early recurrent infections with *Staphilococcus aureus* and *Haemophilus influenzae*, and the stable chronic bacterial infection with *Pseudomonas aeruginosa* (*P. aeruginosa*) that follows in at least 80% of CF teenagers, are not completely understood (9), as ALS dehydration and increased mucus viscosity are considered early predisposing events in CF lung pathophysiology (10, 11).

Hallmarks of the lung pathology of CF patients include defective mucociliary clearance and chronic bacterial infection (especially *P. aeruginosa*) associated with an exaggerated neutrophil dominated inflammation.

## Neutrophils in CF Airway Inflammation: a Double-Edged Sword

Neutrophils are the predominant immune cells infiltrating the airway mucosa and filling the intralumenal space of bronchioles in CF patients (7). Although the recruitment of neutrophils in CF airways' begins early in life and becomes persistent, neutrophils are unable to solve CF bacterial infection. The inefficacy of neutrophils in clearing bacteria prompted a debate on the presence of a neutrophil dysfunction in CF airways, as has been extensively reviewed elsewhere (12, 13). Different *in vitro* and *in vivo* studies in human and mice models evidenced that defective CFTR expressed in CF neutrophils, which is essential for chloride transport into phagolysosome and production of HOCl, impairs bacterial killing, implicating a specific disadvantage in microbial clearance in CF airways (14–18). As an indirect confirmation of the role of CFTR in neutrophilic function, VX-770 CFTR potentiator and VRT-325 corrector partially restored the impaired bacterial killing function in neutrophils of patients bearing G551D-CFTR or F508del-CFTR mutations, respectively (19, 20).

Although defective in clearing the chronic respiratory infection of these patients, neutrophils in CF airways are exposed to bacteria and become a source of continuous release of proteases, mainly elastases, which further impair their killing ability upon cleavage of the CXCR1 chemokine receptor (21). The relevance of elastases released from neutrophils has become an intense field of investigation due to its multiple adverse effects in CF lung pathology. It has been directly correlated with the onset of bronchiectasis and the severity of lung disease. The imbalance between proteases and anti-proteases in the CF ASL has prompted researchers to consider neutrophil elastase as a relevant molecular target in this disease (22–31). Its role in CF lung tissue damage has been further increased by its effect on degradation of CFTR protein (32), which can potentially reduce the efficacy of novel CFTR modulators, and by the evidence that its expression is upregulated by the pro-inflammatory cytokine TNF-alpha (TNF-α) and the chemokine interleukin (IL)-8 (or CXCL8) in CF lung (33). Finally, it amplifies the autocrine circuitry of inflammation by potentiating the recruitment of elastase-producing neutrophils by inducing the release of the neutrophilic chemokine IL-8, acting with an autocrine mechanism on CXCR1 and with activation of TLR4 and MyD88-dependent signaling (34–36).

A second critical adverse effect of a huge amount of neutrophils is their contribution to increasing the pro-oxidant milieu of the CF ALS, as has been extensively reviewed elsewhere (37). Among the different sources of pro-oxidants in the CF airway milieu, neutrophils contribute by releasing reactive oxygen species (ROS) by mechanisms known as “frustrated phagocytosis” or as a result of continuous activation, being the neutrophil-derived ROS critical effectors of bronchial epithelial damage (38–41).

**Figure 1:**
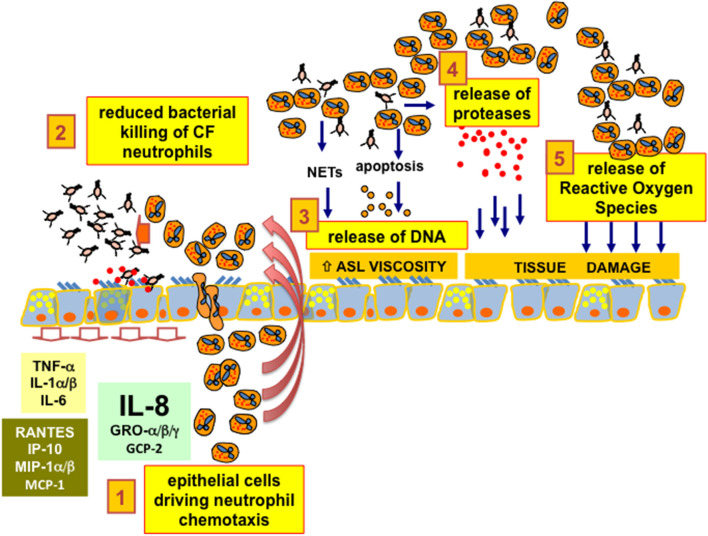
CF bronchial epithelial cells and neutrophil chemotaxis. 1. epithelial cells driving neutrophil chemotaxis: bronchial epithelial cells drive the chemotaxis of neutrophils inside the lumen of bronchi and bronchioles of CF patients, mainly secreting the chemokine IL-8 (CXCL8) expressed upon interaction with bacteria with ASGM1R and TLR2/5 receptors exposed on the apical membrane. 2. reduced bacterial killing of CF neutrophils: CF neutrophils present different degrees of defective killing of the bacteria in the process of phagocytosis, being unable to clear completely the recurrent and, later on, chronic bacterial infection in the airways. 3. release of DNA: CF neutrophils are continuously stimulated in releasing Neutrophil Extracellular Traps. Despite the expected increase in efficiency of bacterial clearing, DNA from NETs increases the viscosity of the Airway Surface Liquid (ASL), further worsening the beat of cilia and the effectiveness of the CF mucociliary clearance. Moreover, CF neutrophils further release DNA as a consequence of apoptosis or necrosis due to the hypoxic environment resulting from mucus plugging of the conductive airways. 4. release of proteases: CF neutrophils exposed to bacteria or bacteria-derived components are prone to exocytosis of granules containing a wide armamentarium of proteases that are usually utilized by neutrophils to disrupt the tissues to allow their migration. In particular, an abundant amount of elastase has been found in CF lungs and its concentration has been related to CF lung disease progression and severity. 5. release of Reactive Oxygen Species: the unbalanced pro-oxidant milieu of the CF ASL due to excessive ROS derives mainly from “frustrated phagocytosis” of CF neutrophils and contributes together with proteases to a neutrophil-dependent airway tissue damage, a clear side effect dependent on the CFTR defect and worsened by an exaggerated neutrophil chemotaxis in CF bronchial lumen.

As a third critical adverse effect, the presence of a large amount of neutrophils in CF brochial lumena implies the release of abundant DNA on the surface of the mucosa, which further reduces the fluidity of the ASL and worsens the bronchial obstruction (42). For a long time, neutrophil-derived DNA was thought to be the result of the turnover of neutrophils ending in hypoxic necrosis and consequent DNA release (43). More recently, the free DNA in CF airways has been found to be derived from the Neutrophil Extracellular Traps (NETs) released by neutrophils instead of the results of hypoxic necrosis (44). NETs are part of the innate defense armamentarium that block bacteria, viruses, and parasites facilitating the phagocytosis by neutrophils. However, in the environment of CF lung infection and inflammation, the benefits of NETs seems to be overcome by the adverse effect of the release of DNA that further reduces ASL fluidity, which impairs the clearance of toxic enzymes, such as neutrophil elastase and myeloperoxidase, damaging the respiratory tissue. The balance between the pros and cons of NETs in CF lung disease is therefore critical (45, 46). A crucial issue in CF lung disease is that neutrophils in the bronchial lumena of CF patients are unable to clear the bacterial infection and are co-responsible, together with bacteria, for the tissue damage, since neutrophils release proteases and ROS and further affect the rheology of CF ASL with abundant DNA. A graphical summary is presented in Figure 1.

## Chemokines Recruiting Neutrophils and the Role of Bronchial Epithelial Cells

Elevated concentrations of cytokines have been found early in life in the bronchoalveolar lavage fluid of CF infants, even in the absence of an overt bacterial infection (4–6). Different mechanisms have been proposed to explain the early onset of sterile inflammation in CF lungs, including the role of airway surface mucus and hypoxia (47–49).

Among the soluble mediators of inflammation detected in CF bronchoalveolar lavage fluid, the most potent chemokines recruiting neutrophils have attracted particular interest, both in terms of pathophysiology and therapeutic perspectives, namely the complement system-derived C5a, the leukotriene B4 (LTB4), and the chemokine Interleukin(IL)-8.

C5a activated complement component receptor (C5aR) expressed on neutrophils was found to be critical in the defense against *P. aeruginosa* infection since knock-out mice deficient of C5aR were able to recruit neutrophils but succumbed to pneumonia because of the killing defect (50). Although it was earlier speculated that the C5aR decoy molecule C5L2 could be beneficial in reducing excessive inflammation in several lung diseases, including CF (51), it was later concluded that inactivation of C5aR by cleavage mediated by proteases released from CF neutrophils was at least partly responsible for the reduced killing and clearance of *P. aeruginosa* in CF lungs (52). Due to this critical role of C5aR in host defense, although elevated concentrations of C5a in CF airway fluids have been directly correlated with disease severity, very little effort has been invested in inhibiting the C5a-C5aR axis (53, 54).

LTB4 is released from neutrophils and macrophages in response to different stimuli, and in turn recruits and activates neutrophils (55). LTB4 has been found at elevated concentrations in different CF respiratory fluids (56–58). Because of its potent neutrophil chemotactic effect, novel drugs, or drug repositioning to inhibit either LTB4 or its receptor have been proposed in CF to reduce inflammatory-dependent tissue damage (59). Particularly relevant was the experience with BIIL 284 BS, a drug acting as an LTB4 receptor antagonist, which was tested in pre-clinical and clinical trials (60, 61). Unfortunately, the clinical trial resulted in major adverse events, as it was apparent that the drug, while effectively reducing the inflammatory response, was untowardly increasing *P. aeruginosa* bacterial load (61, 62), providing a first relevant alert on the delicate balance between the reduction of excessive inflammation by over-inhibiting neutrophil chemotaxis and the mandatory need of preserving a sufficient immune defense. To tackle the crucial issue of anticipating in pre-clinical assays the effect of potential anti-inflammatory molecules modulating neutrophil chemotaxis, an interesting CF *in vitro* model has been set-up, able to simulate closely the transepithelial neutrophil migration and the effect of candidate drugs (63). In spite of inhibiting the LTB4 receptor, such as in the previous unsuccessful experience with BIIL 284 BS (61, 62), acebilistat, a recent drug modulating LTB4 expression, has positively passed pre-clinical assays (63) and is now in clinical trials (64), keeping open the possibility of reducing excessive CF lung inflammation by modulating neutrophil chemotaxis targeting the leukotriene-LTB4 axis.

IL-8/CXCL8 is possibly the neutrophilic chemokine most extensively studied in CF lung pathophysiology. Different cells of the airway tract are known to contribute to the release IL-8; among these, bronchial epithelial cells have been highlighted as a relevant source. To dissect the specific contribution of bronchial epithelial cells in the expression of IL-8 and of several other soluble mediators of inflammation, different *in vitro* experimental models with immortalized cell lines or primary cell cultures have been tested and are currently utilized to investigate molecular mechanisms or novel anti-inflammatory molecules. Key pro-inflammatory challenges able to elicit inflammatory mediators in bronchial epithelial cells are different living bacteria (*S. aureus, P. aeruginosa*), heat-inactivated dead bacteria (Heat-Killed *P. aeruginosa*), flagella-defective or pili-defective recombinant strains of *P. aeruginosa*, single bacterial components (e.g., flagellin), *P. aeruginosa* clinical isolates and bacterial products from patients' airway specimens (e.g., Supernatant of Mucopurulent Material), different pro-inflammatory cytokines (e.g., TNF-α and IL-1), and oxidants such as hydrogen peroxide (65–72). Under these stimuli *in vitro*, bronchial epithelial cells upregulate the basal expression of many pro-inflammatory mediators, such as the cytokines (e.g., TNF-α, IL-1α/β, IL-6), chemokines attracting lympho-monocytes (e.g., IP-10, RANTES, MCP-1, MIP-1α/β), and, as anticipated, IL-8 and other chemokines attracting neutrophils (Gro α/β/γ, GCP-2) (67, 68, 70, 71, 73–81). Among these soluble mediators of inflammation, the neutrophilic chemokine IL-8 (CXCL8) is most strikingly expressed up to two orders of magnitude above the basal release (81). Wide consensus has been reached so far on the role of bronchial epithelial cells as relevant producers of the potent chemokine IL-8, which in turn forwards a strong recruiting soluble signal to neutrophils to reach the lumen of bronchi and bronchioles in the CF mucosa.

## Host-Pathogen Interactions and Intracellular Signaling Modulating IL-8/CXCL8 Expression

A growing series of evidence based on longitudinal clinical investigations of CF patients is building a strong consensus that the inflammatory process could be even more deleterious to CF lung structure and function than the bacterial infection by itself (83) Sterile inflammation in CF lungs has been evidenced based on the presence of elevated concentrations of cytokines in the bronchoalveolar lavage fluid of CF infants of a few months of age (4–6). Airway surface mucus plugging and hypoxia have been proposed to explain the onset of sterile inflammation in CF lungs (47–49). The possible direct contribution of intracellular CFTR protein defects has been proposed (as described later in paragraph 5), together with mechanisms more downstream than the CFTR-dependent ion transport alterations, such as mucus plugging (84). Although inflammation in the absence of detectable bacterial infection has been demonstrated as a likely initiating event, the whole inflammatory process in CF lung is undoubtedly amplified by the occurrence of polybacterial infection. Host-pathogen interactions between bacteria and bronchial epithelial cells in CF have been most extensively studied for *P. aeruginosa*, the pathogen that colonizes CF airways of almost all CF patients. *P. aeruginosa* in a planktonic state interacts through the pili with Asialo-GM1 receptor (AGM1R) on the apical membrane of airway epithelial cells (65). Flagellum-derived flagellin protein also binds to AGM1R, together with Toll-like receptors (TLR) 2 and 5 (85). Flagellin in particular has been found to elicit a strong intracellular pro-inflammatory signaling, as shown by the single purified protein and by recombinant lab strains of *P. aeruginosa*, in different converging studies led by Prince et al. (68, 72, 86, 87). MyD88-dependent signaling downstream TLR2 and TLR5, elicited by *P. aeruginosa*, activates Mitogen-Activated Protein (MAP) Kinases such as MAPK ERK1/2 and MAPK p38, together with ribosomal S6 kinase (RSK)1/2 and heat shock protein (HSP) 27, which are directly involved in inducing the expression of the neutrophilic chemokine IL-8 (88–90). Besides the activation of MyD88-dependent signaling, *P. aeruginosa* is known to also potentiate the expression of IL-8 by a nucleotide-purinergic receptors loop. Interaction of the bacteria with ASGM1R and TLR5 promotes sustained release of ATP as a classical “danger signal” from the apical membrane (85, 91), interacting with purinergic receptors P2Y2R (92). This activates an intracellular calcium signaling (see paragraph 6), in which phospholipase C beta 3 (PLCB3) plays a key role (78, 93).

For transcription factors (TFs) involved in chemokine expression, the promoter elements of IL-8 gene have been widely studied (89, 94, 95). The mapping of the transcription machinery of the IL-8 gene in human bronchial epithelial cells infected with *P. aeruginosa* was studied not only with the aim of understanding the molecular regulation of IL-8 transcription, but also to propose novel anti-inflammatory approaches and to identify potential pharmacological targets. This issue was addressed by investigating the role of TFs on the transcription of the IL-8 gene in human bronchial epithelial cells. Functional assays were based on the transfection of TF decoy oligodeoxynucleotides, designed to interfere with the interaction of the transcription factors nuclear factor-κB (NF-κB), activating protein (AP-1), CAAT/enhancer-binding protein β (C/EBPβ, also known as NF-IL-6), C/EBP homologous protein (CHOP), and cAMP response element binding protein (CREB) with the corresponding consensus sequences identified in the IL-8 promoter. The treatment of target cells with these decoy oligonucleotides reduced the *P. aeruginosa*-dependent transcription of IL-8, suggesting their participation in the transcriptional machinery (89, 94). These conclusions have been recently confirmed and reviewed (95). On the contrary, IL-8 gene expression is repressed by a combination of molecular events that includes: (a) deacetylation of histones, (b) octamer-1 (Oct-1) binding, and (c) active repression by NF-κB repressing factor (NRF). Histone deacetylase-1 (HDAC-1) activity is involved in IL-8 transcription inhibition, as demonstrated by the fact that HDAC1 inhibition derepresses the expression of IL-8, which involves the recruitment of CREB binding protein (CBP)/p300 to the IL-8 promoter, resulting in hyperacetylation of histones and chromatin remodeling, thus counteracting the repression (96, 97). In terms of Oct-1 activity, it has been demonstrated that Oct-1 (the IL-8 repressor) and CCAAT/enhancer-binding protein (C/EBP) (an IL-8 activator) bind to overlapping elements within the IL-8 promoter. The role of Oct-1 as a transcriptional repressor is sustained by experimental evidence that replacing the Oct-1 repressor with C/EBP induces transcription at the IL-8 promoter (98). Similarly, binding of NRF to a negative regulatory element (NRE) in the IL-8 gene promoter (which incompletely overlaps with the NF-κB response element) also represses IL-8 transcription (99). Interestingly, the transcription factors that have been suggested to regulate IL-8 are also involved in regulating the expression of other pro-inflammatory genes, such as GRO-γ, the intercellular adhesion molecule (ICAM)-1, and the cytokines IL-1β and IL-6 (94).

The possible application of transcription factor(s) targeting (for instance, using decoy oligonucleotides) might be a potential therapeutic intervention, especially in the case delivery issues are solved. In this respect, two recently reported studies have focused on nanomaterial-based delivery systems for overcoming limitations associated with clinical applications of decoy oligonucleotides targeting pro-inflammatory transcription factors (such as those targeting NF-kB) (100, 101).

## The Epigenetic Regulation of the Key Chemokines

**Figure 2:**
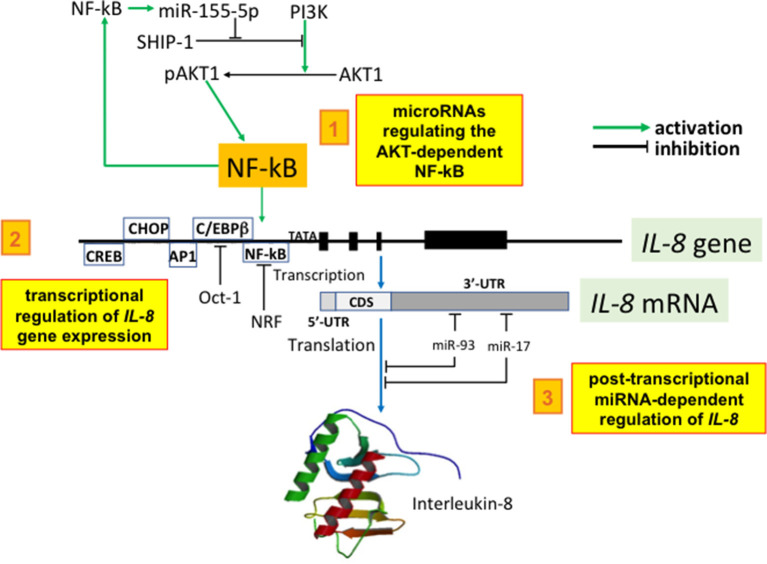
Selected examples of the miRNA network regulating IL-8. 1. microRNAs regulating the AKT-dependent NF-kB: miR-155-5p expression is potentiated by NF-kB and is elevated in CF lung epithelial cells and circulating CF neutrophils biopsied from CF patients. High levels of miR-155 specifically reduce the levels of SHIP1, thereby promoting PI3K/Akt activation. Phospho-Akt levels are therefore elevated in CF lung epithelial cells and can be specifically lowered by either antagomir-155 or elevated expression of SHIP1. Elevated miR-155-5p contributes to the pro-inflammatory expression of IL-8 in CF lung epithelial cells by lowering SHIP1 expression and thereby activating the PI3K/Akt signaling pathway (104). 2. transcriptional regulation of *IL-8* gene expression: *IL-8* promoter is under the control of transcription factors enhancing *IL-8* mRNA production (examples are NF-κB, AP-1, C/EBPβ, CHOP, and CREB); furthermore, IL-8 gene expression is repressed by Oct-1 and by NF-κB repressing factor (NRF). All these transcription factors are regulated by microRNAs. 3. post-transcriptional miRNA-dependent regulation of IL-8: Fabbri et al. identified miR-93 as a miRNA that is decreased in cystic fibrosis IB3-1 and Cufi-1 cells infected with *P. aeruginosa*. The possible involvement of miR-93 in *IL-8* gene regulation was validated using luciferase vectors and the results obtained indicate that IL-8 protein expression is post-transcriptionally regulated by interactions of the *IL-8* mRNA with the inhibitory miR-93 (105). The involvement of microRNAs in IL-8 is also supported by the study of Oglesby et al. (106) who identified miR-17 as a miRNA regulating IL-8. Interestingly, its expression was decreased in adult CF bronchial brushings and bronchial epithelial cells chronically stimulated with *Pseudomonas*-conditioned medium (106). 5′-UTR, 3′-UTR: 5′ and 3′ untranslated region of the IL-8 mRNA; CDS, coding sequences.

The epigenetic regulation of key chemokines in CF occurs at the level of: (a) histone acetyltransferase (HAT)/HDAC balance, (b) histone and DNA methylation, and (c) miRNA-dependent post-transcriptional regulation. It has been recently reported that the transcriptional regulation of IL-8 and other pro-inflammatory genes involves chromatin remodeling through histone acetylation. Interestingly, there is a possible regulatory loop between IL-8 gene transcription and CFTR. In fact, NF-κB facilitates histone acetylation of IL-8 and other pro-inflammatory gene promoters and the histone acetyltransferase (HAT)/HDAC balance is sensitive to CFTR function. This conclusion is supported by the observation that cells with a reduced or absent CFTR function have a decreased HDAC2 protein, resulting in hyperacetylation of the IL-8 promoter and increased IL-8 transcription. In agreement with (a), reduced HDAC2 and HDAC2 activity is observed in cells deficient in CFTR and (b) suppression of HDAC2 expression with HDAC2 shRNA (short hairpin RNA) resulted in enhanced IL-8 expression and promoter acetylation similar to CFTR-deficient cells (102). In conclusion, there is an intrinsic alteration in the HAT/HDAC balance in cells lacking CFTR function *in vitro* and in native CF tissue. This mechanism provides an explanation for the apparent dysregulation of inflammatory mediators seen in the CF airway, as reduced histone deacetylation would potentially influence many genes. IL-8 hypersecretion in CF airway epithelial cells is also caused by the abnormal epigenetic regulation of IL-8 gene involving histone methylation. Under basal conditions, CF cells had increased bromodomain (Brd)3 and Brd4 recruitment and enhanced NF-κB and C/EBPβ binding to the IL-8 promoter compared to non-CF cells due to trimethylation of histone H3 at lysine 4 (H3K4me3) and DNA hypomethylation at CpG6. IL-1β increased NF-κB, C/EBPβ, and Brd4 binding. Furthermore, inhibitors of bromodomain and extra-terminal domain family (BET) proteins reduced IL-8 production in CF cells, suggesting a therapeutic target for the BET pathway (103). Regarding microRNA (miRNA) involved in controlling IL-8 production in the CF lung, three recent studies have determined how miRNAs that are aberrantly expressed in the CF airways may post-transcriptionally regulate IL-8 expression. The first report was focused on miR-155, a miRNA that is highly expressed in CF lung. Bhattacharyya and Coll found that expression of miR-155 was elevated in CF IB3-1 lung epithelial cells in culture, compared with control IB3-1/S9 cells. In addition, clinical evidence indicated that miR-155 was also highly expressed in CF lung epithelial cells and circulating CF neutrophils from CF patients. High levels of miR-155 specifically reduced levels of SHIP1, thereby promoting PI3K/Akt activation and contributing to the pro-inflammatory expression of IL-8 in CF lung epithelial cells (104). The other two studies on this topic have investigated miRNAs that are decreased in the CF lung that directly target IL-8 mRNA. Fabbri et al. identified miR-93 as a miRNA that is decreased in IB3-1 and Cufi-1 cells infected with *P. aeruginosa*. The possible involvement of miR-93 in IL-8 gene regulation was validated using three luciferase vectors, including one carrying the complete 3'-UTR region of the IL-8 mRNA and one carrying the same region with a mutated miR-93 site. Specifically, the results obtained indicate that, in addition to NF-κB-dependent up-regulation of IL-8 gene transcription, IL-8 protein expression is post-transcriptionally regulated by interactions of the IL-8 mRNA with the inhibitory miR-93 (105). The involvement of microRNAs in IL-8 is also supported by the study of Oglesby et al. (106), who measured the expression and function of miRNAs decreased in the CF lung. MicroRNA miR-17 was identified as a miRNA that regulates IL-8 and its expression was decreased in adult CF bronchial brushings and bronchial epithelial cells chronically stimulated with *Pseudomonas*-conditioned medium (106). Another microRNA involved in inflammation is miR-636, as recently demonstrated by Bardin et al. (107). By analyzing miRNAs in human primary air-liquid interface cell cultures, overexpression of miR-636 in CF patients compared to non-CF controls was shown. Functional studies demonstrated that miR-636 directly interacts with IL1R1 and RANK (two pro-inflammatory cytokine receptors), and IKBKB (which encodes IKKβ, a major protein in the NF-κB pathway). A summary of miRNA regulation is depicted in Figure 2.

## CFTR Protein Defects Cooperate to Pro-inflammatory Intracellular Signaling

The origin of this abnormal inflammatory response in CF continues to be debated, with researchers unsure whether it is initiated by exogenous stimuli, such as persistent microbial infection, or by intrinsic deficiency of CFTR function, with alterations in signal transduction, or both. Dysregulation of the airway innate immune system is associated with CF, being both airway epithelial cells and immune cells susceptible to intrinsic CFTR-associated alterations in signal transduction.

No differences in TLRs expression have been observed between human CF and non-CF airway epithelial cell lines. However, increased expression of TLR2 and TLR5 on the apical membrane of the polarized human CF airway epithelial cells and in CFTR-knockout vs. WT mice were found (108, 109). These different cellular redistributions of TLRs render the CF airway epithelial cells more susceptible to host-pathogen interactions between bacterial constituents (pili and flagellin) and the receptors involved in the transduction of “danger signal” at apical membrane, favoring the release of cytokines (as described before in section host-pathogen interactions and intracellular signaling modulating IL-8/CXCL8 expression).

An intrinsic defect associated with CFTR deficiency is the susceptibility of CF airway cells to accumulate ROS, particularly during pathogen infection. Evidence shows increased oxidative stress in CF (37, 110–116). Although several studies suggest that the oxidative stress in CF is a direct consequence of mitochondrial dysfunction due to perturbed CFTR signaling, others suggest that the accumulation of ROS in CF depends on there being a reduced antioxidant capacity (113). Low mitochondrial reduced glutathione (mtGSH) levels were found in CF patient-derived tracheal cells and in CFTR-knockout mice (117, 118). Indeed, in CF patient-derived pancreatic and tracheal cells a reduction of protein expression of Cu/Zn-superoxide dismutase (SOD1) and Mn-SOD (SOD2) and a reduction in the activity of extracellular SOD (119) has been observed. The oxidative stress in CF lung may: (I) affect autophagy, compromising the expression of CFTR channel (120, 121); (II) induce mtDNA oxidation and damage, triggering inflammasome activation, and/or altering the OXPHOS activity, that in turn produces additional ROS (122, 123); and (III) destroy lung tissue, affecting the cell function and/or exacerbating the inflammatory response (37).

An inherent defective CFTR channel leads to transglutaminase (TG2) upregulation, resulting in defective autophagy with consequent accumulation of aggresomes and ROS (121). Lack of autophagy in macrophages and in airway epithelial cells result in a reduced bacterial clearance and in the accumulation of dysfunctional mitochondria, which in turn promotes secretion of pro-inflammatory cytokines, indicating that autophagy may regulate the inflammation responses by suppressing the secretion of immune mediators (124–126). In fact, the rescue of dysfunctional autophagy in CF, mediated by autophagy inducers such as MTOR inhibitor (rapamycin), TG2 inhibitor (cistamine), and/or modulators of Ca^2+^-dependent signaling (KB-R7943), attenuated the hyperinflammation in CF lung, improving the CFTR transport to PM and reducing ROS production and cytokine release in macrophages and in primary CF airway cells *in vitro* and in CF mouse models *in vivo* (127–132).

The hyperinflammation of the CF airway is sustained by the accumulation of dysfunctional mitochondria, as an indirect consequence of perturbed CFTR signaling, responsible for ROS production and increased inflammasome-dependent IL-1β release. Mitochondrial defects associated with an abnormal oxidative stress and inflammatory response have been found in CF. The first evidence was shown in 1979, demonstrating that mitochondrial Ca^2+^ uptake and oxygen consumption were altered in mitochondria isolated from CF patient-derived fibroblast (133). In the same year, the mitochondrial NADH dehydrogenase (complex-1) was found altered in CF skin fibroblasts (134), while in 1981 a deficiency of 6-phosphate dehydrogenase in CF patients was found (135). Consistent with the first observations, in CF patient-derived tracheal cells a decreased mitochondrial NADH dehydrogenase activity and mitochondrial membrane potential (Δψ) were measured, due to the down-regulation of the mt-ND4 gene that codifies for a subunit of the complex-1 essential for its assembly and activity (136). This alteration was rescued through the reintroduction of CFTR-wt, also indicating that mtGSH depletion in CF is responsible for the altered complex-1 activity (118). Recently, it has been observed that the CFTR corrector agent VX-809 and 4,6,4'-trimethylangelicin (TMA) treatment lead to partial restoration of the mitochondrial failure in CF airway cell lines, producing an improvement in complex-1 activity, Δψ generation, ANT-dependent ADP/ATP exchange, and membrane lipid peroxidation (137). These data indicate that the restoration of mitochondrial physiology is linked to the mitigation of the inflammatory response, suggesting that the mitochondrial defects found in CF airway cells contributes to the susceptibility of CF cells to bacterial infection, influencing the innate immune response.

Several pieces of evidence have shown how the hyperinflammation observed in CF lung is sustained by cell defects associated with CFTR deficiency intrinsic to epithelial, but also in inflammatory, cells. In CF patients, the differentiation of T lymphocytes to Th_17_ phenotype is increased (138), while monocyte-derived macrophages do not respond to IL-13/IL-4 and fail to polarize into M2 while the polarization to the M1 phenotype was unaffected (139). CF macrophages serve as a replicative niche for bacteria to avoid host defenses (140), present deficits in bacterial killing (141, 142), and produce excess cytokines (143). In synthesis, although the initiating event of CF lung inflammation between infection-derived exogenous and CFTR-related endogenous components remains a “chicken and egg” debate still without a final consensus, different converging evidence supports the hypothesis that CFTR-specific signal transduction alterations amplify the extent of the response.

In this context, analysis at a single-cell level might be very informative. For instance, Mould et al. (144) investigated inflammatory macrophage heterogeneity during acute lung inflammation in mice and performed single cell RNA sequencing of macrophages isolated from the airspaces during peak inflammation and resolution of inflammation. They found two transcriptionally distinct subdivisions of alveolar macrophages based on proliferative capacity and inflammatory programing. Of course, overcoming technical obstacles (due, for instance, to debris, and apoptotic cells) rendering difficult isolation of individual cells for single cell analyses is a key issue (145). Finally, on-a-chip devices are expected to bring key information at single-cell levels on hyperinflammation in pulmonary diseases, including CF (146–148). As far this key issue is concerned, starting from the notion that cell-to-cell variability in chemokine/cytokine secretion is largely unknown, Ramji et al. developed and validated a microfluidic device to integrate live-cell imaging of fluorescent reporter proteins with a single-cell assay of protein secretion (146). This device was used to image transcription factor dynamics in macrophages in response to LPS, followed by quantification of secretion of TNF, CCL2, CCL3, and CCL5.

## Intracellular Calcium Homeostasis in the Regulation of the Expression of Chemokines

Ca^2+^ homeostasis is a pivotal element in regulating the immunological and physical barriers of the airway epithelium in CF. Normally, the exposure to common respiratory bacterial pathogens, such as *P. aeruginosa*, originates in a cytosolic Ca^2+^ transient (about 100 nM) in airway epithelial cell lines necessary to initiate the inflammatory response, inducing the expression, and secretion of pro-inflammatory cytokines (149). A defective CFTR channel leads to the deregulation of Ca^2+^ homeostasis in CF cells, which is detrimental for lung inflammation. Ca^2+^ signaling dysregulations were observed in several human CF airway epithelial cell lines, where the intracellular Ca^2+^ concentration is increased compared to non-CF cells (150) due to: (I) intrinsic defects associated with CFTR deficiency, (II) chronic exposure to bacterial infection, and (III) persistent stimulation by pro-inflammatory mediators.

### Intrinsic Defects Associated to CFTR Deficiency

In 1961, Donnell et al. published the first evidence of alterations of Ca^2+^ homeostasis in CF patients (151), which was then confirmed by Feigal et al., through direct measurement of the increased intracellular Ca^2+^ concentration in fibroblasts derived from CF patients (152, 153) and by Cabrini and De Togni as increased cytosolic Ca^2+^ concentration in CF neutrophils (154). In 1982, an increased mitochondrial Ca^2+^ uptake was observed in CF skin fibroblast, due to altered respiratory system activity (155). In 2009, Antigny et al. measured a decreased mitochondrial Ca^2+^ uptake in F508delCFTR airway epithelial cell lines as a consequence of depolarized and fragmented mitochondria (156). The debate is presently lessened because it has been demonstrated that a functional CFTR channel reduces the basal intracellular Ca^2+^ concentration in human airway epithelial cell lines and donor-derived primary airway epithelial cells, influencing the mitochondrial Ca^2+^ signals evoked by physiological and pathological stimuli (132). This abnormal intracellular Ca^2+^ increment in CF epithelial airway cells is in part justified by the reduced activity of Plasma Membrane (PM) Ca^2+^ ATPase (PMCA), which limits the Ca^2+^ efflux through the PM, and increased SERCA activity, which favors the endoplasmic reticulum (ER) Ca^2+^ accumulation (132). This increment in intracellular Ca^2+^ concentration in human epithelial airway cells is normalized by the administration of the corrector agent, VX809 (157). Several other pieces of evidence suggest that the increased intracellular Ca^2+^ concentration observed in CF airway cells depends on multifactorial aspects associated with defective CFTR, involving many Ca^2+^ channels expressed in the PM, including: (I) the Transient Receptor Potential Canonical channel 6 (TRPC6), normally expressed in human primary CF epithelial cells. Its Ca^2+^ influx capacity is enhanced in F508delCFTR and G551D-CFTR cells (158, 159); (II) the Store Operated Ca^2+^ Entry (SOCE) resulted in significantly increased CF airway cell lines and primary cells, due to an enhanced Orai1 channel insertion to PM, with consequent exacerbation of IL-8 secretion (160); and (III) the TRP channel TRPA1, expressed in bronchial columnar epithelial cells. Its direct activation by *P. aeruginosa* increases Ca^2+^ entry, mediating the release of cytokines such as IL-8, IL-1β, and TNF-α (81).

### Chronic Exposure to Bacterial Infection and Persistent Stimulation by Pro-inflammatory Mediators

The chronic infection of CF airways amplifies the altered intracellular Ca^2+^ homeostasis of CF epithelial cells, predisposing the airway cells to a hyperinflammatory profile, which contributes to producing an excess of cytokines. Bacterial constituents and pro-inflammatory mediators cooperate, inducing an abnormal intracellular Ca^2+^ signaling in CF airway epithelia due to increased activation of apical G protein-coupled receptors (GPCRs) and a sustained ER Ca^2+^-release (161). The higher intracellular Ca^2+^ concentration in CF cells contributes to a greater and more prolonged NF-κB activation with consequent effects on the expression and release of pro-inflammatory cytokines, such as IL-8 and IL-1β (82, 161). The persistent NF-κB activation in human CF airway cells is the consequence of the synergistic effects of bacterial components, such as flagellin, where flagellin interacting with asialoGM1 receptor favors the release of ATP from CF airway cell lines, which mediates purinergic receptors and activates downstream intracellular Ca^2+^ signaling that synergizes with the TLR5-dependent signaling to activate NF-κB (see section host-pathogen interactions and intracellular signaling modulating IL-8/CXCL8 expression) (92). The release of nucleotides from bronchial epithelial cells targeting P2Y2 purinergic receptors has been proposed to intervene on different aspects of CFTR regulation and lung pathophysiology (162–165). Bacterial constituents, pili and flagellin, interact with TLRs and TLR-associated glycolipid in airway cells (85, 166). In particular, TLR2 or asialoGM1 linked to TLR2 express both on the apical surface of airway cells and recognize bacterial constituents to induce the pro-inflammatory transcription of CXCL8 or MUC-2 gene via NF-κB activation, through the recruitment of PI3K and phospholipase C gamma (PLCγ), which in turn stimulate the release of Ca^2+^ through Inositol Triphosphate Receptors (IP3R) channels (167, 168). The generation of cytosolic Ca^2+^ transients activates classical Protein Kinase C (PKC) α and β isoforms, which through a phosphorylation cascade mediate the activation of NF-κB (169). In a similar molecular pathway, PLC beta 3 (PLCB3) also plays a relevant role in triggering cytosolic Ca^2+^ transients induced by *P. aeruginosa*, regulating the activation of PKCα and PKCβ to induce an NF-κB-dependent transcription of CXCL8 gene in human airway epithelial cell lines and in patient-derived primary cells (78). β-sitosterol (BSS) was used to inhibit the active form of PKCs involved in the transduction of *P. aeruginosa*-dependent pro-inflammatory Ca^2+^-dependent signaling in CF patient-derived airway epithelial cells, leading to a significant reduction in expression of IL-8, growth-related oncogene (GRO)-α, and GRO-β (170). The role of PLCB3 in amplifying the expression and release of IL-8 during pathogen infection, through the regulation of intracellular Ca^2+^ transients, is associated with the severity and progression of CF lung disease. Single Nucleotide Polymorphisms (SNP) genetic study with the progression of CF lung disease severity, identify from a panel of 135 genes of immune response the association of c.2534C>T (p.S845L) variant of PLCB3 with a mild progression of pulmonary disease in CF (93). PLCB3-S845L results in a loss-of-function variant, where defective intracellular Ca^2+^ redistribution and PKCs' activation limited the IL-8, IL-1β, and MUC5 expression in CF patient-derived airway epithelial cells exposed to *P. aeruginosa* or CF patient-derived mucopurulent material. The IP3R-mediated ER Ca^2+^-release is significantly augmented in CF epithelial cell lines (171), a consequence also of ER Ca^2+^ store expansion observed in CF cells (150). The ER expansion is not dependent on ER retention of misfolded CFTR, but reflects an airway epithelial response acquired following persistent bacterial infection, resulting in ER unfolded protein response (UPR) activation mediated by the IRE1/XBP-1 pathway and in a larger intracellular Ca^2+^ mobilization in response to abnormal GPCRs activation (172).

The sustained ER Ca^2+^-release in CF airway cells conditions the mitochondria to a direct involvement in the pro-inflammatory response. CF airway cell lines and CF patient-derived airway primary cells are prone to *P. aeruginosa*-dependent mitochondrial perturbations, in which the mitochondrial Ca^2+^ uniporter (MCU) is a signal-integrating organelle that mediates mitochondrial ROS-dependent NLRP3 inflammasome activation and recruitment of both NLRP3 and NLRC4 inflammasome (132). The degree and quality of the inflammatory response in CF airway cells is also sustained by *P. aeruginosa*-dependent mitochondrial perturbations, initiated by flagellin, such as mitochondrial membrane potential loss, ROS production, and mitochondrial fragmentation (132). Rimessi et al. have characterized the role of mitochondria as drivers of the *P. aeruginosa*-triggered inflammatory exacerbation in CF airway cells, demonstrating that mitochondrial Ca^2+^ signaling plays a critical role in inflammasome NLRP3 recruitment and inflammasome-dependent IL-1β and IL-18 release in CF airway cell lines and in CF patient-derived airway primary cells (132). By modulating the MCU-dependent mitochondrial Ca^2+^-uptake, genetically or mediating pharmacological inhibition with KB-R7943, it is possible to control the pathogen-dependent mitochondrial dysfunction preventing the integration of pro-inflammatory signals from mitochondria into CF patient-derived airway primary cells and *in vivo* mouse models (131, 132).

Although whether the Ca^2+^-dependent activation of chloride channels in CF bronchial epithelial cells could partially vary the defects of CFTR ion transport is presently under scrutiny (173), the results recalled above support the concept that the up-regulation of intracellular Ca^2+^ signaling is a key amplifier of the inflammatory response and lung pathogenesis in CF, which opens the issue of new potential molecular therapeutic targets.

## Targeting Neutrophil Chemotaxis in CF: Novel Molecules and Drug Repurposing

Preliminary observations in rat lung models (59) suggested the repurposing of ibuprofen, a non-steroideal anti-inflammatory drug used in conditions like osteoarthritis, rheumatoid arthritis, juvenile idiopathic arthritis, and acutely painful musculoskeletal conditions, to clinical use for CF patients (174, 175). Long-term application of this drug, as reported by a Cochrane analysis, has proved the concept that strategies to modulate lung inflammation can be beneficial for people with CF (176). As ibuprofen inhibits prostaglandin synthesis (177), a very broad anti-inflammatory mechanism that is not closely specific to the pathophysiology of CF lung inflammation, innovative approaches to target the adverse effects produced by the huge amount of neutrophils in the CF conductive airways have been recently tested. Some of these approaches have been launched upon the knowledge of the specificity of CF lung inflammation, whereas others were just pure empirical testing. The first attempts were focused on antagonizing neutrophil proteases elastase, one of the main deleterious effect of neutrophil inflammation in CF (28, 29, 178–186). Different elastase inhibitors have also been recently tested in clinical trial, with promising results in terms of safety and tolerability (187, 188), maintaining the high levels of interest in the rationale of targeting neutrophil elastase in CF lung inflammation (186).

Early signaling evoked by bacteria has been tested by inhibition of TLR2 or by inducing extracellular calcium entry through calcium ionophores (81, 189). More downstream the production of IL-8, different antagonists of its receptors that block its action on cell targets have been challenged (190–192). More recently, different molecular approaches targeting the intracellular signaling in bronchial epithelial cells have been developed. In consideration of the key role of NF-κB in the transcriptional regulation of IL-8 and other pro-inflammatory genes, several studies have been focused on pharmacological alteration of NF-κB activity. The transcription factor (TF) decoy strategy was applied by Bezzerri et al., using TF oligodeoxynucleotides (ODNs) to NF-κB able to inhibit transcription of IL-8 in bronchial cells (89). The TF decoy approach was based on the intracellular delivery of double-stranded ODNs causing inhibition of the binding of TF-related proteins (as determined *in vitro* using EMSA assays) to the different consensus sequences in the promoter of specific genes. When CF cells were transfected with double-stranded TF “decoy” ODNs, mimicking different NF-κB consensus sequences, partial inhibition of *P. aeruginosa*-dependent transcription of IL-8 was obtained. In addition, other NF-κB regulated genes were inhibited, such as GRO-gamma and IL-6. In order to demonstrate that TFD against NF-κB interferes with the NF-κB pathway, Finotti et al. demonstrated mediating chromatin immunoprecipitation (ChIP) treatment with TFD oligodeoxyribonucleotides of IB3-1 cells infected with *P. aeruginosa* leads to a decreased occupancy of the IL-8 gene promoter by NF-κB factors (193). Further studies were focused on the development of more stable therapeutic molecules and on the delivery strategy for TFD molecules. Among stable ODN analogs, peptide nucleic acids (PNAs)-based agents were found to be promising for CF. In this respect, PNA-DNA-PNA (PDP) chimeras are molecules of great interest from several points of view: (a) they can be complexed with liposomes and microspheres; (b) they are resistant to DNases, serum, and cytoplasmic extracts; and (c) they are potent decoy molecules (194, 195). By using electrophoretic mobility shift assay and RT-PCR analysis, it was demonstrated that: (a) the effects of PDP/PDP NF-κB decoy chimera on the accumulation of pro-inflammatory mRNAs in *P. aeruginosa*-infected IB3-1 cells in particular; (b) the PDP/PDP chimera is a strong inhibitor of IL-8 gene expression; and (c) the effect of PDP/PDP chimeras, unlike those of ODN-based decoys, are observed even in the absence of protection with lipofectamine (193–196). In another study, NF-κB decoys were employed with the hypothesis that they may limit lung inflammation in CF. In the study by De Stefano et al. (197), the effects of decoy ODN targeting NF-κB and delivered through biodegradable and respirable poly(D,L-lactide-co-glycolide) large porous particles (LPP) were determined on IL-6 and IL-8 mRNA expression in CF cells stimulated with lipopolysaccharide (LPS) from *P. aeruginosa*. The conclusion was that respirable biodegradable decoy ODN LPP may represent a promising strategy for inhibiting NF-κB transcriptional activity and related gene expression. This treatment, *in vivo*, was expected to reduce lung chronic inflammation in CF patients. Interestingly, De Stefano et al. (198) investigated the effects of NF-kB decoys delivered with inhalable nanoparticles in a rat model of lung inflammation induced by intratracheal aerosolization of LPS from *Pseudomonas aeruginosa*. A single intratracheal insufflation of the decoy ODNs reduced the bronchoalveolar neutrophil infiltration induced by LPS. This reduction was associated with decreased NF-κB/DNA binding activity, and decreased the content of IL-6, IL-8, and mucin-2 in lung homogenates.

In consideration of the involvement of microRNAs in the post-transcriptional regulation of IL-8 and other pro-inflammatory genes, both antagomiR and miRNA replacement approaches have been proposed (199–202). This confirmed that, in addition to relevance for the theoretical point of view, the studies on epigenetic regulation of chemokines (described in Chapter 5) might be important for the development of therapeutic protocols. For instance, transfection of CF cells with miR-93 (105) and miR-636 mimics (107) leads to an IL-8 decrease (105) and to a reduction of NF-κB activity, causing decreased secretion of IL-8 and IL-6 (200, 201). Another miRNA target to be considered is miR-199a-3p, whose expression is inversely correlated with increases in the expression of IKKβ and IL-8 (200). On the other hand, targeting miR-155 with antagomir might also be considered for IL-8 reduction (104). In fact, down-regulation of miR-155 was found to suppress the IL-8-associated pro-inflammatory phenotype in CF cells. In order to reduce the miR-155 levels in CF cells, antagomiR molecules against miR-155 were employed, modified with cholesterol to permit efficient entry into cells. Incubation of IB3-1 cells with antagomir-155 effectively down-regulated miR-155 expression, together with a sharp decrease in IL-8 mRNA and protein levels (104). On the other hand, as expected, miRNAs were also demonstrated to directly target the 3′UTR of IL-8 mRNA, such as miR-93 (105) and miR-17 (105). Therefore, modulating the expression of miRNAs that target IL-8 mRNA in CF bronchial epithelial cells is likely to represent a new therapeutic strategy for CF (199–201).

These studies have conclusively demonstrated that the pro-inflammatory status in CF is under the control of a complex network constituted by transcription factors and non-coding RNAs, responsible for transcriptional and post-transcriptional regulation of the expression of genes, such as IL-8, belonging to the pro-inflammatory CF network. These studies allowed the identification of novel targets for pharmacological interventions based on newly designed therapeutic approaches.

In addition to the development of new experimental approaches, recent efforts have been undertaken on drug repurposing, in order to bring new therapies based on drugs already used for other indications. This is expected to bring to the market several treatments at a lower risk, reduced cost, and less development time when compared to conventional drug development programs (202–204). One of the most interesting classes of molecules are psoralens, extensively studied as molecules to be employed in PUVA (Psoralen and Ultraviolet A)-therapy, a treatment extensively used in a variety of pathological conditions, including eczema, psoriasis, graft-vs.-host disease, vitiligo, mycosis fungoides, large-plaque parapsoriasis, and cutaneous T-cell lymphoma (205). It was in a study that found that 5-methoxypsoralen reduces *P. aeruginosa*-dependent IL-8 transcription in bronchial epithelial cell lines (206). When the analysis was extended to analogs of 5-methoxypsoralen (207, 208), a potent effect was observed with 4,6,4′-trimethyl-angelicin (TMA), which inhibited *P. aeruginosa*-dependent IL-8 transcription at a nanomolar concentration in IB3-1, CuFi-1, CFBE41o-, and Calu-3 bronchial epithelial cell lines. Analysis of phosphoproteins involved in pro-inflammatory transmembrane signaling evidenced that TMA reduces the phosphorylation of ribosomal S6 kinase-1 and AKT2/3, which were found to be involved in *P. aeruginosa*-dependent activation of IL-8 gene transcription (208). In addition, to understand whether the NF-κB pathway should be considered a target of TMA, chromatin immunoprecipitation was performed, demonstrating that TMA (100 nM) preincubated in whole living cells reduced the interaction of NF-κB with the promoter of IL-8 gene. These results suggest that TMA could inhibit IL-8 gene transcription mainly by intervening on driving the recruitment of activated transcription factors on the IL-8 gene promoter, as demonstrated in NF-κB (208). Recently, TMA was also shown to exhibit, in addition to anti-inflammatory activity, potentiation and correction of the CFTR. In conclusion, TMA is a triple-acting compound that reduces excessive IL-8 expression and potentiating/correcting CFTR function (209, 210). Another repurposed drug proposed for possible anti-inflammatory effects is azithromycin (AZM). IL-8 expression and DNA binding activity of two key pro-inflammatory transcription factors, NF-κB and AP-1, were investigated in CF and isogenic non-CF airway epithelial cell lines. AZM reduced both IL-8 mRNA and protein expression in CF cells reaching the levels of non-CF cells. In the presence of AZM reduction of NF-κB and AP-1, DNA binding was also observed (211). Regarding anti-inflammatory approaches, *in vitro* studies have tested the effects of genistein, fluvastatin, and corilagin, amongst others (212–214). The isoflavonoid genistein [5,7-Dihydroxy-3-(4-hydroxyphenyl)chromen-4-one] reduces IL-8 production in cultured CF bronchial gland cells by increasing cytosolic IκBα protein levels, thereby inhibiting NF-kB activation (212). The statin fluvastatin [(±)-(3R′,5S′,6E)-7-[3-(4-Fluorophenyl)-1-isopropylindol-2-yl]-3,5-dihydroxy-6-heptenoate] decreased IL-8 production in whole blood in response to *Pseudomonas* or *Aspergillus* antigens, by preventing the prenylation of molecules, such as rho-A, ras, or rac, implicated in IL-8 signaling (213). Corilagin [beta-1-O-galloyl-3,6-(R)-hexahydroxydiphenoyl-d-glucose], a gallotannin identified in several plants, including *Phyllanthus urinaria*, binds to NF-κB, thus inhibiting NF-κB/DNA interactions and decreasing IL-8 gene expression in CF bronchial IB3-1 cells (214).

As for drug repurposing, corilagin, already shown to exhibit versatile medicinal activities, was found to be of potential use as a possible therapeutic molecule for CF. Interestingly, in addition of IL-8 inhibition, corilagin inhibits TNF-α-induced secretion of MCP-1 and RANTES (214).

The possible identification of repurposed drugs was also tackled by alternative approaches, such as connectivity mapping (ssCMap) to predict A20-inducing drugs and their anti-inflammatory action in CF. A20 is a NF-κB down-regulator that is expressed at low levels in CF and it is hypothesized to be a key target to normalize the inflammatory response (215). Publicly available gene array expression data, together with a statistically significant connections' map (sscMap), were employed. The objective was to predict drugs already licensed for therapeutic use in human pathologies to induce A20 mRNA and protein expression and thereby reduce inflammation. Ikarugamycin and quercetin have been identified as possible candidates for anti-inflammatory approaches, analyzing their effects on A20 and NF-κB(p65) expression (mRNA) as well as IL-8 pro-inflammatory cytokine release in the presence and absence of bacterial LPS in bronchial epithelial cells lines and in primary nasal epithelial cells from patients with CF and non-CF controls (215). Despite the very interesting results obtained from studies of drug repurposing in CF, the safety assessment in a new disease indication (CF in this case) is still an important concern in the regulatory process. While the safety assessment is based on drug label information, the drug repurposing approach may involve different formulations, changes in dosage that should be given great attention in the different patient populations considered.

## Concluding Remarks and Perspectives

The present review outlines different specific pathophysiological aspects of CF lung inflammation in which the bronchial epithelial cells represent a “crossroad of signaling” in this disease. Particular emphasis has been given to the role of bronchial epithelial cells in driving the process of neutrophil chemotaxis, with special regard to intracellular signaling, that could be considered a therapeutic target to reduce the lung tissue damage dependent on the byproducts released by hyper-activated neutrophils in the CF bronchial mucosa. To translate into therapy, a special focus was placed on innovative molecules or on drug repurposing to target pathways that are specific of the CF lung pathophysiology, instead of testing broad range anti-inflammatory molecules. As CF lung inflammation is a clearly secondary effect of altered CFTR protein defective ion transport, a question arises on whether in the era of CFTR modulators, with increasing efficacy in CFTR rescue, gating potentiation, and PM stabilization, anti-inflammatory drugs maintain a specific therapeutic rationale. It has already been shown that rescue of F508del CFTR in CF in experimental model systems can partly reduce the release of pro-inflammatory mediators, including IL-8 (216–218). However, the CFTR correctors and potentiators are not available for all the classes of CFTR molecular defects, leaving a significant fraction of CF patients without this treatment option. The clinical response has been shown to be variable within patients with the same class of mutations; the advanced inflammatory disease in adolescent and adult CF patients is unlikely to be completely halted using only CFTR correctors and potentiators, leaving the development of novel anti-inflammatory drugs a rational unmet need for CF treatment (219, 220).

## Author Contributions

GC, AR, PP, and RG initiated the concept and wrote the manuscript together with MB, IL, and AF. All authors contributed to the article and approved the submitted version.

## Conflict of Interest

The authors declare that the research was conducted in the absence of any commercial or financial relationships that could be construed as a potential conflict of interest.
